# Total circulating microRNA level as an independent prognostic marker for risk stratification in breast cancer

**DOI:** 10.1038/s41416-022-01756-z

**Published:** 2022-03-22

**Authors:** Aoife Ward Gahlawat, Lavinia Fahed, Tania Witte, Sarah Schott

**Affiliations:** Department of Gynecology and Obstetrics, University Hospital of Heidelberg, Heidelberg, Germany

**Keywords:** Breast cancer, Prognostic markers

## Abstract

**Background:**

Although breast cancer (BC) has a high survival rate, relapse events may occur which ultimately lead to aggressive disease. Circulating cell-free microRNAs (cf-miRNAs) are a promising minimally invasive biomarker with diagnostic and/or prognostic potential. Unfortunately, there is still no consensus as to a universal cf-miRNA biomarker in BC and there has been no clinical implementation until now. One major limitation is the technical variation with cf-miRNA isolation and specific quantification methods.

**Methods:**

In this study, we assessed the total levels of cf-miRNAs as a potential prognostic marker for BC in 356 plasma samples from 250 BC patients.

**Results:**

High levels of cf-miRNAs significantly correlated with unfavourable clinical features including tumour stage, load and the presence of metastasis at diagnosis. With more than 9 years of follow-up, we could show that global cf-miRNA levels significantly correlated with cancer relapse which was confirmed in multivariate cox regression analysis. Finally, for a subset of patients where the serial plasma was available, levels of cf-miRNAs increased in the plasma prior to clinical detection of progressive disease and were massively elevated in patients who died compared to those still alive at the last timepoint of measurement.

**Conclusions:**

This is the first study to suggest that total cf-miRNA levels in the blood can be used as an independent prognostic marker for BC.

## Introduction

Breast cancer (BC) alone accounts for 30% of all new cancer diagnoses in women. While the majority of cases are diagnosed with localised disease, with a 5-year survival rate of up to 99%, it is estimated that more than 40,000 American women will die from aggressive and metastatic disease in 2020 [1]. After surgery and curative treatment, women with BC are monitored by clinical examination and imaging. Due to the varying levels of sensitivity and specificity with these methods, the presence of metastasis or relapse might go unnoticed at a routine check-up. Additional biomarkers for aggressive BC are desirable to guide interventions and to reduce overdiagnosis and over- or under-treatment of this common, but complex disease.

In the past decade, circulating nucleic acids have been explored as potential liquid biopsy markers for cancer detection, disease monitoring and prognosis. This area of translational research has great potential to complement current diagnostic methods for cancer patients. One study found that elevated circulating tumour DNA (ctDNA) levels in BC preceded clinical detection of metastasis in 86% of patients with an average lead-time of 11 months and that the quantity of ctDNA correlated with poor survival [2]. Yet the implementation of ctDNA as a clinical biomarker for BC poses a number of limitations. The downstream methods such as sequencing and digital PCR are costly and technically challenging as well as a large amount of plasma required for such assays [3].

Circulating cell-free microRNAs (cf-miRNAs) are emerging as a new class of promising minimally invasive clinical biomarkers for profiling of cancer patients [4, 5]. miRNAs are short RNA species (typically 22 nucleotides in length) that regulate gene expression and may have tumour suppressive or oncogenic functions in cancer [6–8]. Cf-miRNAs in the bloodstream may originate from apoptotic or necrotic cells. In cancer, it has been hypothesised that cf-miRNAs are released from tumours in microvesicles such as exosomes [5]. In ovarian cancer for example, a panel of exosomal miRNAs were found to be significantly correlated with primary tumour miRNA expression compared to women with benign disease and could not be identified in healthy controls [9]. On the contrary, other studies have suggested that cf-miRNAs originate from blood cells rather than being tumour specific [10]. Regardless of their origin, cf-miRNAs have been shown to be remarkably stable in body fluids such as plasma, which gives them great potential as a non-invasive diagnostic tool for early cancer detection [11–14]. In contrast to ctDNA, they are relatively easy to obtain and measure from minute amounts of blood.

While many extraction and quantification methods are available, there is still no standard processing protocol for cf-miRNAs, which has hindered their clinical implementation. For BC alone, the list of potential cf-miRNAs as biomarkers continues to grow with each lab using different methods leading to conflicting results [15]. In addition, qPCR standardisation is challenging and no robust reference controls for data normalisation have been established until now [16]. Moreover, different extraction protocols and lab variations lead to method dependent bias affecting qPCR outcomes [17]. The clinical application of cf-miRNA requires standardisation, which could be achieved with a simple straightforward measurement of total cf-miRNAs.

In this study, we evaluate whether plasma cf-miRNA levels at diagnosis can differentiate among [1] clinical characteristics of BC (stage, tumour size, grade, metastasis) and [2] women diagnosed with BC who will relapse and/or die from the disease. In a subset of patients, we evaluate plasma cf-miRNA levels in longitudinal samples and correlate this to disease outcomes.

## Methods

### Ethics approval and consent to participate

Breast cancer (BC) patients were recruited at the Women’s Hospital, University Heidelberg, Germany between 2009–2014, prior to surgery and chemotherapy. All patients provided written informed consent to participate in the study. This study was approved by the ethics committee of the University of Heidelberg (S-039/2008, S-684/2018) in accordance with good clinical practice guidelines, national laws, and the Declaration of Helsinki.

### Sample collection

The patients filled in a questionnaire on sociodemographic information and whole-blood samples were collected. Patient characteristics are summarised in Table 1. From each patient, three EDTA tubes (Sarstedt S-Monovette K3E, 1.6 mg EDTA/ml) with 9 ml of whole blood were taken.

**Table 1:** BC patient characteristics.

	*n*	%
BC patients	250	100
Age (mean)	51	(26–87 years)
Follow-up time (median)	4.9	(0–9.4 years)
Subtype		
Luminal A	107	42.8
Luminal B	80	32
Her2+	32	12.8
TNBC	31	12.4
Stage		
1	92	36.8
2	112	44.8
3	20	8
4	22	8.8
Unknown	4	1.6
Grade		
1	31	12.4
2	147	58.8
3	71	28.4
Unknown	1	0.4
Tumour size		
<2 cm	101	40.4
2–5 cm	113	45.2
5+ cm	32	12.8
Unknown	4	1.6
Lymph node		
N0	180	72
N1	60	24
Unknown	10	4
Treatment		
Radiotherapy	184	73.6
Chemotherapy	165	66
Endocrine therapy	173	69.2
Recurrence or metastasis		
Yes	77	30.8
No	173	69.2
Survival status		
Dead	38	15.2
Alive	212	84.8

### Plasma preparation

Whole-blood samples were centrifuged at 1300 × *g* for 20 min. The plasma fraction was further processed by high-speed centrifugation at 12,000 × *g* for 10 min. Samples were immediately snap-frozen and stored at −80 °C.

### miRNA isolation from plasma

I Circulating miRNAs were isolated from 300 µl thawed plasma using the NucleoSpin miRNA Plasma kit (Macherey-Nagel) according to the manufacturer’s protocol. Total miRNAs were quantified using the Qubit microRNA Assay Kit and the Qubit Fluorometer 3.0 (Thermo Fisher Scientific). Plasma cf-miRNA levels are presented as ng/µl.

### Data analysis

Clinical data were curated with Microsoft Excel. The BC subtype was defined by histopathology reports. Luminal B tumours were assigned when Ki67 staining was >20% based on current diagnostic standards at the time of diagnosis. For comparison of patient characteristics to cf-miRNA levels, a Fisher’s exact test was used for categorical variables. Kaplan–Meier survival plots for survival were computed with GraphPad prism Version 8, using the Log-rank model for significance. Follow-up dates were calculated from the date of diagnosis until any event (relapse, death or last known follow-up). Patients were dichotomised according to the median cf-miRNA amount (<0.6 vs. >0.6 ng/ul). In order to compute univariate hazard ratios, a Cox proportional hazards regression model [18] was fitted for each parameter separately with the respective overall survival or progression-free survival time as a dependent variable and the respective marker as a single covariate. Multivariate hazard ratios are stemming from a multivariate Cox proportional hazards regression model, where all markers were simultaneously used as covariates when fitting the Cox models. For all Cox models, *P* values for the null hypothesis that the hazard ratio equals to 1 were derived by means of a standard Wald test. Univariate and multivariate analyses were carried out in SPS software. The REMARK (Reporting Recommendations for Tumour Marker Prognostic Studies) guidelines were implemented to report results [19].

## Results

### Patient cohort

**Fig. 1:**
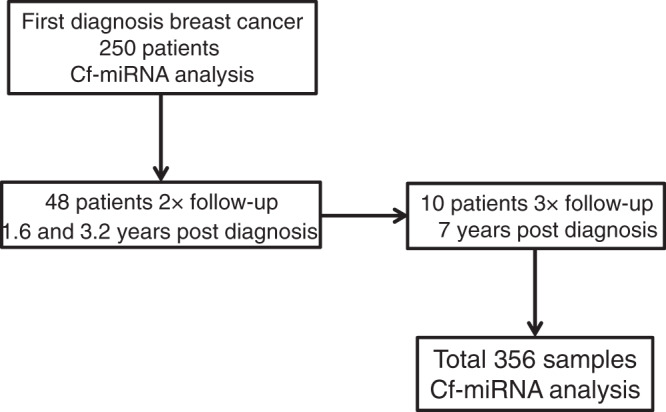
Overview of the study. Samples from 250 women diagnosed with BC were analysed in this study. For 48 of the patients, an additional 2 follow-up samples were obtained and for 10 of the patients, a third follow-up was available.

We prospectively evaluated a population of 250 BC patients treated at the University Hospital Heidelberg, Germany. Patients were enrolled in the study upon the first presentation of a breast lesion between August 2009 and August 2014. The presence of cancer was confirmed by a tumour biopsy. The cohort had a mean age of 51 (Table 1). The majority of patients (*n* = 204) presented with Stage 1 or Stage 2 cancer. Just 20 patients presented with Stage 3 and 22 with Stage 4 disease. Follow-up information was collected up to 9.4 years post the first diagnosis with an average of 4.8 years, in which time 38 patients died from their disease and 77 had a relapse event. For 48 of the patients, serial blood samples were taken at follow-up appointments (average 1.6 and 3.2 years after cancer diagnosis) for longitudinal monitoring. For a subset of these patients, a third sample at an average of 7 years post diagnosis was available for analysis (Fig. 1). Clinical relapse was determined via imaging and/or clinical examination.

### Higher cf-miRNA levels correlate with adverse tumour characteristics

The total amount of cf-miRNA was measured in plasma samples collected at BC diagnosis (*n* = 250). Cf-miRNA amounts ranged from 0 to 7.5 ng/µl per patient with a median 0.6 ng/µl (equal to 60 ng total) in the cohort (Supplementary Fig. 1). Patients were dichotomised according to the median into either “low” or “high” cf-miRNA groups. The clinicopathological characteristics according to cf-miRNA levels are presented in Table 2. Higher levels of cf-miRNA at diagnosis were significantly associated with advanced age at diagnosis (*P* < 0.0001), advanced tumour stage (*P* = 0.0006), the presence of local (*P* = 0.0116) or distant metastasis (*P* = 0.0006) and higher tumour grade (*P* = 0.0021) (Table 2). In addition, there was a highly significant association with cf-miRNA levels and patients who either relapsed or died (Table 2).

**Table 2:** Clinicopathological characteristics of patients according to cf-miRNA levels.

		Low cf-miRNA		High cf-miRNA		
		*n*	%	*n*	%	*P*
Age	<50	82	32.8	53	21.2	
	>50	38	15.2	77	30.8	**<0.0001**
Stage	1 and 2	108	43.9	96	39	
	3 and 4	10	4	32	13	**0.0006**
Size	T1–2	107	43.5	107	43.5	
	T3–4	11	4.4	21	8.5	0.1288
Nodal status	N0	98	40	89	36	
	N1–3	20	8	40	16	**0.0116**
Metastasis	M0	114	46.5	109	44.5	
	M1	3	1.2	19	7.7	**0.0006**
Grade	G1	23	9.2	8	3.2	
	G2–3	97	38.9	121	48.6	**0.0021**
Relapse (Stage 1–3)	Yes	15	6.5	43	18.8	
	No	102	44.7	68	29.8	**<0.0001**
Death (all stages)	Yes	5	2	33	13	
	No	115	46	97	39	**<0.0001**

*P* values were obtained using Fisher’s exact test for categorical factors.

Significant *P* values are shown in bold.

### Total cf-miRNA is an independent prognostic marker for risk stratification in breast cancer

**Fig. 2:**
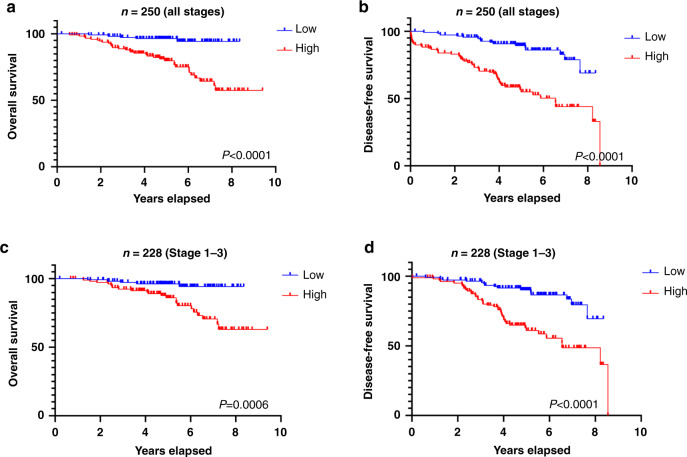
Cf-miRNA levels associate with OS and DFS. Patients were dichotomised based on the median cf-miRNA level and associations with overall survival (**a**) and disease-free survival (**b**) were depicted after Kaplan–Meier survival analysis. The same analysis was performed, excluding patients with Stage 4 disease (**c**, **d**). *P* values shown represent a statistical significance between the two curves.

To investigate whether cf-miRNA levels had a prognostic impact in BC, Kaplan–Meier analysis for overall survival (OS) and disease-free survival (DFS) was performed. Higher levels of baseline cf-miRNA predicted patients at high risk of both death and relapse (Fig. 2a, b). Since metastasis has a huge impact on patient survival, we repeated this analysis excluding patients with Stage 4 disease. High levels of cf-miRNA significantly associated with OS and DFS in the reduced cohort (Fig. 2c, d). To prove that these results were specific for cf-miRNA levels, we subsequently performed univariate and multivariate Cox regression analysis including patient age, cancer subtype, stage and grade as outcome variables. Total cf-miRNA levels were not significant for OS in both univariate and multivariate analyses but were highly significant for PFS in both settings (Table 3, multivariate HR = 1.2 CI [1.01–1.41] *P* = 0.03). When we performed the above analysis using dichotomised cf-miRNA levels, high cf-miRNA was an independent predictor of both OS and PFS in both univariate (Table 3) and multivariate analysis (Supplementary Table 1).

**Table 3:** Univariate and multivariate analysis.

	Univariate HR	95% CI	*P* value	Multivariate HR	95% CI	*P* value
Overall survival						
Age	1.05	1.02–1.08	0.0005	1.04	1.01–1.07	0.006
Subtype	1.48	1.19–1.84	0.0004	1.60	1.21–2.12	0.001
Stage	2.39	1.73–3.31	1.22E-07	2.51	1.70–3.71	3.77E-06
Grade	2.00	1.12–3.57	0.0184	1.34	0.65–2.76	0.435
cf-miRNA all	1.15	0.93–1.42	0.1951	0.98	0.69–1.39	0.921
cf-miRNA median	8.04	2.8–22.8	0.0001			
**Progression-free survival**						
Age	1.04	1.02–1.07	0.00015	1.03	1.01–1.05	0.009
Subtype	1.21	1.02–1.45	0.03278	1.19	0.97–1.47	0.099
Stage	3.64	2.73–4.86	1.48E-18	3.21	2.35–4.38	2.29E-13
Grade	1.61	1.05–2.48	0.03039	1.34	0.78–2.32	0.294
cf-miRNA all	1.32	1.16–1.50	0.00002	1.20	1.01–1.41	0.035
cf-miRNA median	6.58	3.21–13.48	2.64E-07			

**Fig. 3:**
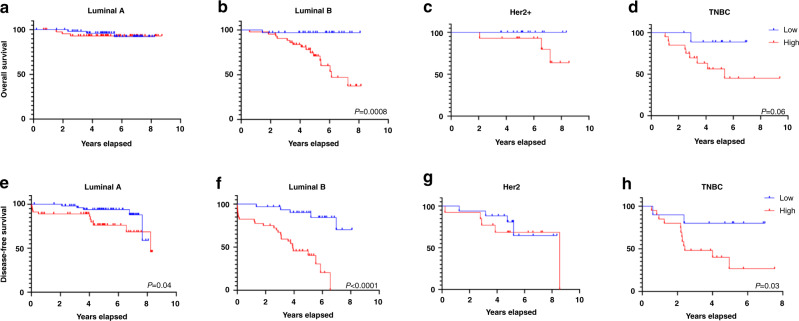
OS and DFS prediction based on cf-miRNA levels according to tumour subtype. Patients were dichotomised based on the median cf-miRNA level and further split into groups dependent on the breast cancer subtype. Associations with overall survival (**a**–**d**) and disease-free survival (**e**–**h**) were depicted after Kaplan–Meier survival analysis. *P* values shown represent a statistical significance between the two curves.

Because BC is a very heterogeneous disease, we next sub-classified the survival analysis based on the tumour subtype. Baseline levels of cf-miRNAs had no impact on OS of Luminal A patients, where almost no events occurred (Fig. 3a). High levels of cf-miRNAs significantly associated with OS in Luminal B patients (Fig. 3b). For Her2+ and TNBC, high levels of cf-miRNA were not statistically significant for OS (Fig. 3c, d). On the other hand, cf-miRNA levels were significantly associated with shorter DFS in Luminal A, Luminal B and TNBC subtypes, but not in Her2+ patients (Fig. 3e–h). Taken together, these results indicate that cf-miRNA levels are highly predictive of the risk of tumour relapse and disease progression.

### Disease surveillance using serial cf-miRNA analysis

**Fig. 4:**
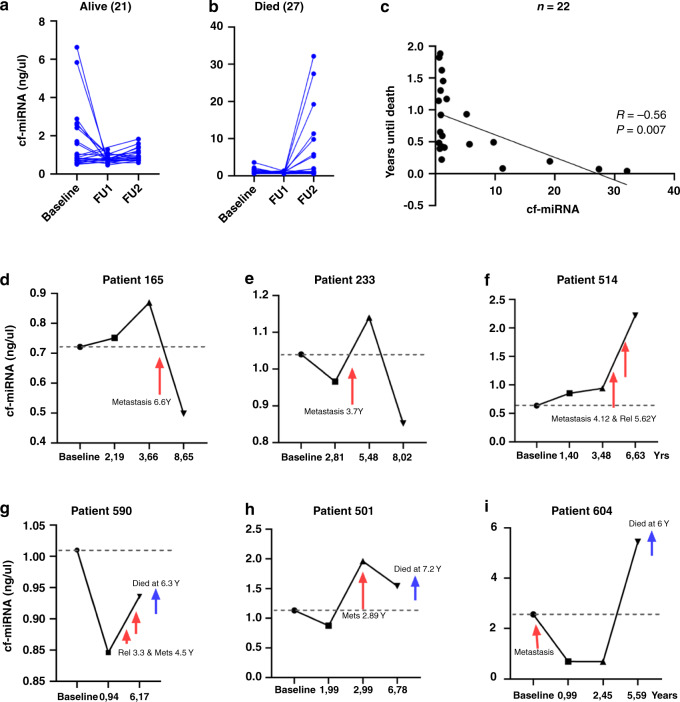
Serial blood analysis reveals the potential of cf-miRNA in disease monitoring. Cf-miRNA level in longitudinal samples of patients who were still alive (**a**) or who died from their disease (**b**) and the association between cf-miRNA and the years until death (**c**). Specific follow-up times and cf-miRNA levels for six patients in the cohort (**d**–**i**).

Next, we examined longitudinal cf-miRNA levels in a subset of 48 patients where samples were available. At the last known timepoint (average 7 years), 27 of the patients had died from BC and 21 were still alive. In addition to the baseline sample, two additional samples (average time 1.6 and 3.2 years post diagnosis) were collected. For those who were still alive, levels of cf-miRNA reduced and stabilised at the second timepoint compared to the baseline (Fig. 4a). In contrast, for the patients who died, cf-miRNA levels increased as the disease progressed (Fig. 4b). Furthermore, we found a significant inverse correlation between levels of cf-miRNA and time until death in patients where we had a sample within 2 years of their death (Fig. 4c). For ten patients, we could obtain a third follow-up sample at ~7 years post diagnosis (Fig. 1). Next, we zoned in on the individual patient profiles of three patients who were still alive (Fig. 4d–f) and three who since died (Fig. 4h–i).

For patients 165 and 233, with stable disease, cf-miRNAs reduced to levels below the baseline. While in the additional patients with progressive disease, cf-miRNA levels were elevated at the last timepoint. In addition, the elevation of cf-miRNAs preceded the clinical detection of relapse in all of the patients observed (Fig. 4d–i). Although we could only follow a subset of patients, we believe that this data supports the idea that cf-miRNA levels are predictive of disease outcome which might provide great added value to current diagnostics. In conclusion, high cf-miRNA levels are an accurate and independent blood-based predictor for poor survival and relapse in BC.

## Discussion

Our results show that total cf-miRNA levels are an independent prognostic marker in breast cancer (BC). In our cohort, the baseline amount of cf-miRNA could discriminate between two groups of patients with different outcomes when followed for more than nine years. To our knowledge, this is the first study exploring the quantification of cf-miRNA levels as a predictive marker in BC. We believe that our novel findings can serve as a hypothesis generator, supporting the utility to implement cf-miRNA as a predictive tool in larger randomised trials and open options for individualised care strategies.

There is a need to better stratify BC patients into different risk groups more accurately than can be achieved with current clinicopathologic classification methods. With better stratification, low-risk patients could be spared unnecessary treatment, avoiding side effects and reducing the cost of treatment. For example, it has been shown that adjuvant chemotherapy is not beneficial to most women with BC [20]. On the other hand, patients at high risk can be more closely monitored and receive timely treatment. Liquid biopsy markers have great potential as we approach the era of individualised treatment strategies.

Our work highlights that baseline cf-miRNA levels could predict those patients at the highest risk of relapse and has therefore potential to inform current therapy decisions and improve survival statistics. If BC is localised in the breast, the 5-year survival rate is up to 99%. However, metastases in distant organs and/or disease recurrence are major events impacting survival. Current clinical monitoring lacks the sensitivity to detect such events. The implementation of an additional biomarker that could identify such high-risk patients is highly desirable in times of more favourable therapeutic options than conventional chemotherapies. Not only could we show that baseline cf-miRNA levels associate with patients at the highest risk of relapse but higher levels of cf-miRNAs preceded the clinical diagnosis of metastases, relapse and death.

While many recent studies have focused on ctDNA and had similar results to us, we believe that cf-miRNA level is much more advantageous as a liquid biopsy marker. Here, we used the straightforward Qubit instrument to quantify cf-miRNAs. Although this method may not be as sensitive as sequencing or PCR, it can be easily adapted to the clinical setting. The assay is simple, affordable, quick and can complement current standard methods. Secondly, work from our group and others has proven that cf-miRNA is highly stable in blood which has been stored for up to 1 week [14], frozen for up to 17 years [21] and across different extraction protocols. In addition, the majority of cf-miRNA studies have implemented qPCR as a detection method and focused on specific miRNAs, which in itself has a lot of technical variability. Another advantage of Qubit is the little amount of blood required for analysis. Compared to ctDNA assays, requiring upwards of 2 ml plasma, cf-miRNA can be done with ten times less plasma. In line with another recent study [17] column-based extractions are highly effective for isolation of cf-miRNAs. We have used the Machery–Nagel kit in our study and this is about half the price of similar kits on the market. In conclusion, measuring cf-miRNA levels is very cost-effective and time-saving in comparison to any methods for cf-DNA.

High levels of cf-miRNA at diagnosis are associated with a number of adverse clinical characteristics. In particular, cf-miRNAs were significantly elevated in patients with advanced Stage 4 disease. This finding is in line with the current hypothesis that circulating nucleic acids are mostly released from aggressive, late-stage tumours, honing in on the use of liquid biopsy for disease monitoring rather than early detection. Higher cf-miRNA was also significantly associated with advanced age. Interestingly cf-DNA has been investigated as a potential marker of ageing [22]. Our longitudinal analysis in a subset of patients highlighted the potential of cf-miRNA as a prognostic biomarker. Most surprisingly, in women where a relapse event occurred, cf-miRNA levels were already elevated before the diagnosis. Although this result could only be shown in a few patients, we believe it strengthens the data and confirms the prognostic potential of cf-miRNA in BC and warrants further evaluation.

Multivariate Cox regression analysis confirmed that cf-miRNA levels are an independent predictor of PFS but not OS when analysed as a numerical variable. However, when we analysed based on the median cut-off it was highly significant for both OS and PFS. This finding further strengthens our reasoning for choosing the median cf-miRNA level as a cut-off since the majority of patients with low cf-miRNA had a more favourable clinical picture and outcome. In view of clinical implementation, further studies should address if only patients with a “high” value should be more closely monitored.

Our analysis indicates that the addition of cf-miRNA as a biomarker, could help to better stratify patients at high risk of relapse or death. This finding has significant translational relevance, particularly for the TNBC and Luminal B subtypes with the worst prognosis [23], where optimal treatment strategies are still under exploration. Interestingly, for the clinically “good” Luminal A tumours, there was still a significant difference in DFS based on cf-miRNA levels. While these tumours are typically managed by endocrine therapy, the risk of relapse may be better assessed with the addition of cf-miRNA at diagnosis and warrants further evaluation in prospective studies to stratify those tumours and treatment strategies i.e. benefits from prolonged anti-hormonal treatment. For Her2+ subtypes, we did not observe any significant trend which might be due to the low numbers of patients and the fact that they are generally well managed by Her2-targeted therapy. In addition, the women in our cohort have had different treatment strategies over inclusion time which might have affected the outcome compared to worldwide percentages [24].

A major limitation of our work is the lack of validation in an independent cohort. Unfortunately, we did not have access to an additional panel of blood samples, but we would like to confirm our findings in future work. Here, all samples were collected and processed at one centre which reduces variability, yet limits our findings. The results should be reproduced in a larger scale, multicenter studies. Another constraint is that pan cf-miRNA as a biomarker lacks specificity. While our findings suggest that cf-miRNA is a prognostic marker for BC, we have not yet explored other cancer types or diseases. At this point, we can only hypothesise that cf-miRNAs are tumour specific, as others suggest that they originate from blood cells [10]. Finally, lifestyle factors such as sports and food intake have not been taken into account, which may have affected the circulating miRNA profile [25, 26].

In conclusion, this is the first study that implements total cf-miRNA level as a liquid biopsy marker for independent prediction of BC relapse and survival. This intriguing observation should be validated in independent cohorts and across different cancer types.

### Reporting summary

Further information on research design is available in the Nature Research Reporting Summary linked to this article.

## Supplementary information

Supplementary Data

Reporting Summary
