# Supplementary material for: Total circulating microRNA level as an independent prognostic marker for risk stratification in breast cancer
Source: Br J Cancer. 2022 Mar 22;127(1):156–62. doi: 10.1038/s41416-022-01756-z (PMC9276748; doi:10.1038/s41416-022-01756-z)
Supplement: Supplementary file 1 — Supplementary Data [file 41416_2022_1756_MOESM1_ESM.pptx]

## Slide 1
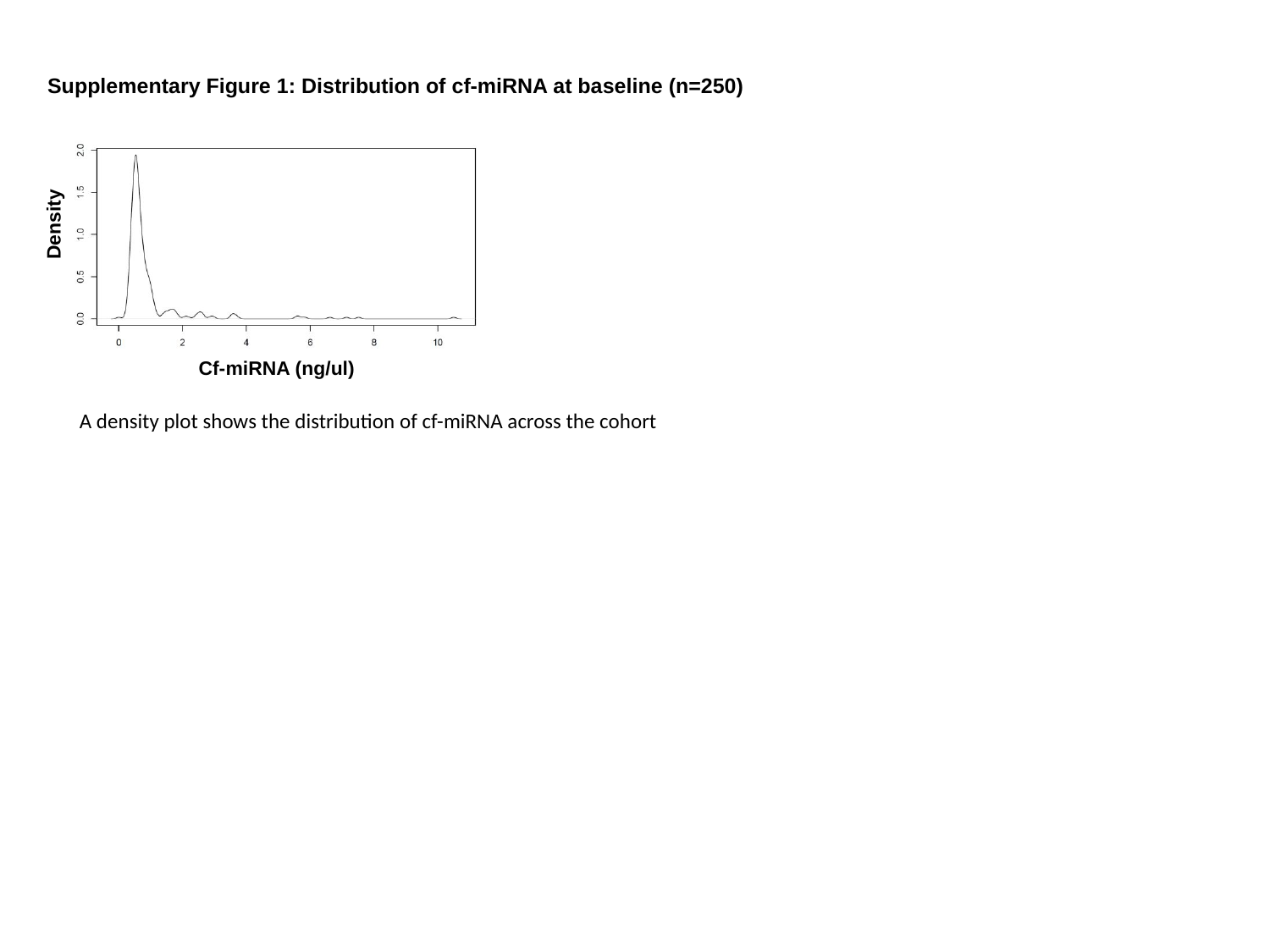

Supplementary Figure 1: Distribution of cf-miRNA at baseline (n=250)
Density
Cf-miRNA (ng/ul)
A density plot shows the distribution of cf-miRNA across the cohort

## Slide 2
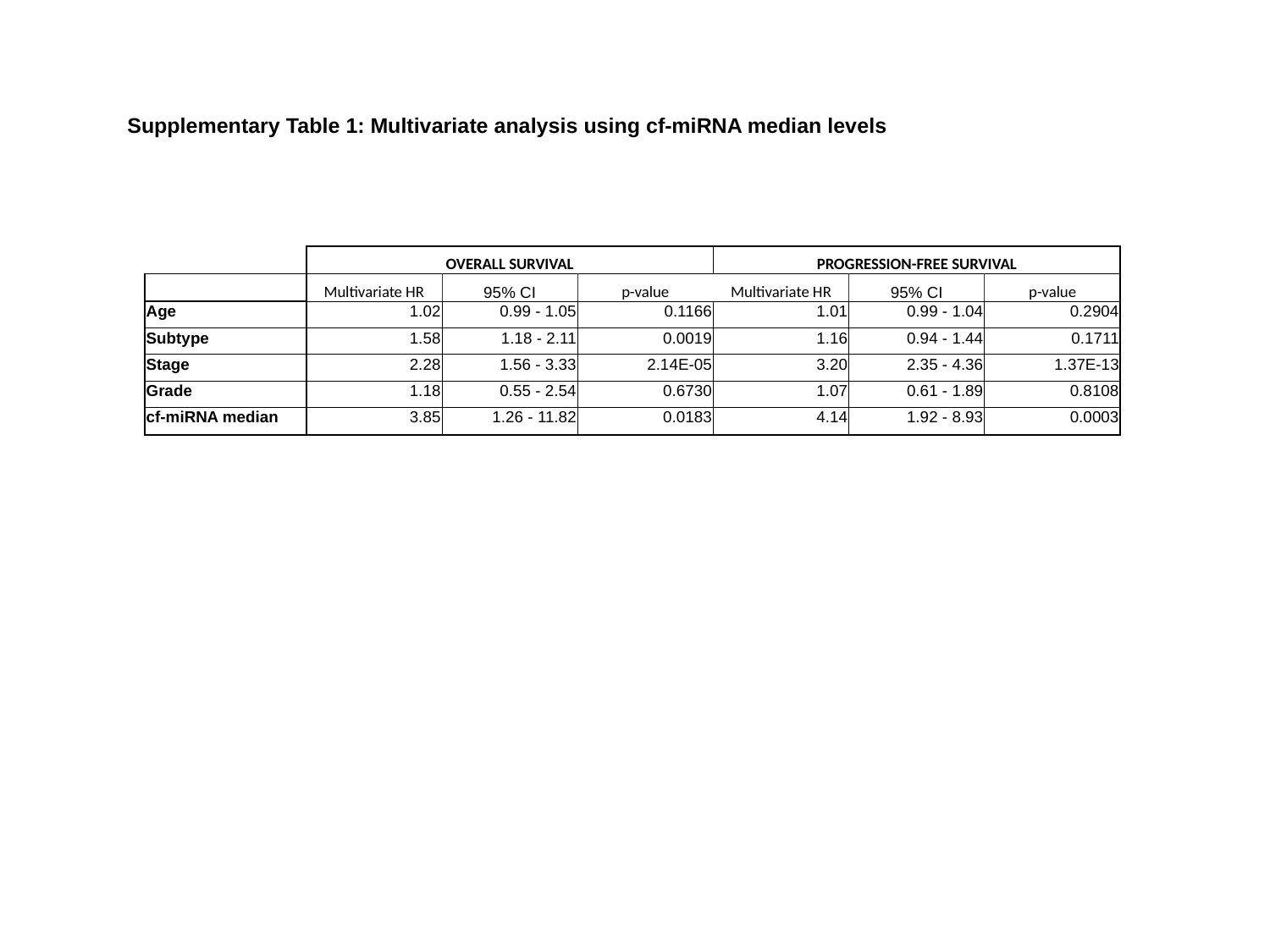

Supplementary Table 1: Multivariate analysis using cf-miRNA median levels
| | OVERALL SURVIVAL | | | PROGRESSION-FREE SURVIVAL | | |
| --- | --- | --- | --- | --- | --- | --- |
| | Multivariate HR | 95% CI | p-value | Multivariate HR | 95% CI | p-value |
| Age | 1.02 | 0.99 - 1.05 | 0.1166 | 1.01 | 0.99 - 1.04 | 0.2904 |
| Subtype | 1.58 | 1.18 - 2.11 | 0.0019 | 1.16 | 0.94 - 1.44 | 0.1711 |
| Stage | 2.28 | 1.56 - 3.33 | 2.14E-05 | 3.20 | 2.35 - 4.36 | 1.37E-13 |
| Grade | 1.18 | 0.55 - 2.54 | 0.6730 | 1.07 | 0.61 - 1.89 | 0.8108 |
| cf-miRNA median | 3.85 | 1.26 - 11.82 | 0.0183 | 4.14 | 1.92 - 8.93 | 0.0003 |
